# Freeze-all policy for *in vitro* fertilization in women with normal response to ovarian stimulation

**DOI:** 10.31744/einstein_journal/2021AO6290

**Published:** 2021-09-30

**Authors:** Oscar Barbosa Duarte-Filho, Sérgio Podgaec

**Affiliations:** 1 Hospital Israelita Albert Einstein São PauloSP Brazil Hospital Israelita Albert Einstein, São Paulo, SP, Brazil.

**Keywords:** Fertilization *in vitro*, Ovulation induction, Embryo transfer, Cryopreservation, Vitrification

## Abstract

**Objective:**

To answer the question if the freeze-all strategy and subsequent frozen embryo transfer is preferable to fresh embryo transfer for patients with normal response to ovarian stimulation (4 to 15 oocytes recovered) during *in vitro* fertilization treatments.

**Methods:**

A retrospective cohort from two human reproduction centers between 2013 and 2017. A total of 471 frozen embryo transfers from freeze-all cycles, and 3,208 fresh transfers were included.

**Results:**

After propensity score matching adjustment for age and number of eggs, 467 freeze-all cycles and 934 fresh cycles were analyzed, revealing no statistically significant difference between groups in relation to clinical pregnancy rate (32.5% in the Freeze-all Group and 32.3% in the Fresh Group, p=0.936). For women aged 40 years and older, we observed a statistically significant higher clinical pregnancy rate when freeze-all strategy was used (29.3% in the Freeze-all Group and 19.8% in the Fresh Group, p=0.04).

**Conclusion:**

Freeze-all strategy was not superior to fresh transfer for all patients with normal response to ovarian stimulation. However, women aged 40 years and older could benefit from this strategy. This deserves further investigation in future research, preferable in a prospective randomized study.

## INTRODUCTION

The first human birth from *in vitro* fertilization (IVF) occurred from a fresh-embryo transfer, in England, in 1978.^( 1 )^ Five years later, an Australian group reported the first birth after frozen embryo replacement.^( 2 )^ Since then, more than seven million babies have been born through IVF with fresh or frozen embryo transfer.^( 3 )^

During the early era of IVF, fresh-embryo transfer was the standard of care, because results following embryo freezing by the slow cooling technique were unsatisfactory.^( 4 )^ Frozen transfers were restricted to surplus embryos and where fresh transfer was not possible, which was the case mostly in patients with a high response to controlled ovarian stimulation, and at risk of ovarian hyperstimulation syndrome.^( 5 )^

In the last decade, the use of thawed embryos has increased significantly. A recent online study, involving experts from several countries, revealed that almost 85% of clinics included in the study routinely offer frozen embryo transfer to their patients.^( 6 )^

Two facts have changed the practice of fresh transfers: the demonstration that replacement of fresh embryos into the endometrium, while under the effects of drugs for ovarian stimulation can alter endometrium receptivity,^( 7 )^ and the development of vitrification methods for human embryos-ultra-fast freezing that is simpler, has better survival and pregnancy results compared with slow cooling.^( 8 )^ These factors have led to a growing debate as to whether the standard of care should shift from the current “freeze-all for selected patients” to a “freeze-all for all” approach.^( 9 - 11 )^

Two recent meta-analyses found similar pregnancy rates between the two strategies for patients with normal response to ovulation stimulation, but there is much heterogeneity among the studies, and the evidence to support this strategy is still considered of low-quality.^( 12 , 13 )^

## OBJECTIVE

To compare clinical pregnancy rates of fresh transfer and frozen embryo transfer in freeze-all cycles in patients who had normal response to ovarian stimulation, and to identify in this set of patients the clinical variables that are associated with higher pregnancy rates within each strategy.

## METHODS

### Patients and cycles

This is a retrospective study including data extracted from electronic medical records of all autologous IVF cycles, which were carried out at the ALFA Project and *Vidabemvinda* clinics, São Paulo, Brazil, between January 2013 and December 2017. Four to 15 mature oocytes were collected (considered the normal response). Only the first transfer for the Fresh Group and the first thaw for the Freeze-all Group were considered for analysis. The exclusion criteria were ovum pick-up without transfer or thaw within the study period; patients involved in the shared oocyte donation program, transfers of embryos accumulated from different egg collections; or cycles undergoing pre-implantation genetic tests (obligatory freeze-all for both clinics). The study and all of its protocols were approved by the Ethics Committee of *Hospital Israelita Albert Einstein* (HIAE) in São Paulo (Brazil) at the time of data collection (approval number: 2.373.642, CAAE: 79213617.1.0000.0071) and of *Hospital Santa Paula* (approval number: 2.506.239, CAAE: 79213617.1.3001.5670) and exempted us from having to apply the informed consent. Confidentiality and integrity of the data were maintained, and patients’ identities were preserved.

### Stimulation protocol and endometrial priming

Controlled ovarian stimulation was performed with recombinant follicle-stimulating hormone, menotropin, or a combination of both. A gonadotrophin-releasing hormone (GnRH) antagonist was used for pituitary suppression in more than 90% of cycles. In all cases with fresh transfer, the trigger included the use of recombinant or urinary human chorionic gonadotrophin (hCG), either alone or in combination with GnRH agonists, according to physician’s choice. Endometrial priming for frozen-embryo transfer was achieved with estradiol and progesterone or with natural cycle, according to the preference of both physician and patient.

### Embryo culture and cryopreservation

Intracytoplasmic sperm injection or conventional IVF was performed in virtually all cases. Embryos were cultured until transfer at the cleavage stage (second or third day) or blastocyst (fifth or sixth day), and were classified according to the description of the Society for Assisted Reproductive Technology (SART).^( 14 )^ In the Freeze-all Group, all embryos were frozen at cleavage or blastocyst stage, using vitrification method with two commercial vitrification kits (Cryotop^®^ Vitrification, Kitazato, Japan; Vitringa^®^, Ingamed, Brazil).

### Variables and outcomes

The independent variables analyzed were female age, number of oocytes and number of matures oocytes, fertilization rate, number of zygotes, number and quality of cleavage embryos and blastocysts and number and stage of the embryos transferred.

The primary outcome was clinical pregnancy, defined as visualization of the gestational sac in uterus on ultrasound scan. If more than one gestational sac was seen, it was considered a multiple pregnancy. Implantation rate was calculated by dividing the number of gestational sacs by the number of embryos transferred. Miscarriage was defined as the interruption of pregnancy after visualization of the gestational sac in the uterus.

### Statistical analysis

We verified the normality of quantitative variables using histograms, box plot and quantile-quantile plots. For the normal quantitative variable, we evaluated the means and standard deviations (SD) and used the Student’s *t* test to compare the means. For non-normally distributed data, we used the median and interquartile range, and employed the Mann Whitney test for comparisons. For qualitative variables, the number and proportion of observations in each category are presented, and data were compared using the χ^2^ or Fisher’s exact test. To evaluate the effect of the fresh and freeze-all approaches on the outcomes of interest, controlling for the other variables, we used generalized linear models of the binomial family with logistic regression (logistic regression models).

We evaluated the balance of samples of the complete database by calculating standardized differences and using the Omnibus χ^2^ test. Based on the results of the simple logistic regression, we paired the groups using the propensity score method considering two covariates: number of mature eggs and female age. Applying this, we matched cases with a ratio of 1:2 of cycles in the Freeze-all and Fresh Groups. This selection of cases was carried out using the nearest-neighbor method; for each transfer in the Freeze-all Group we selected two transfers in the Fresh Group, with the closest propensity score. Results for cleavage and blastocyst transfers were analyzed separately.

## RESULTS

### Cohort characteristics

The present study included 3,679 IVF cycles: 471 that used freeze-all strategy and 3,208 fresh transfers. Of these IVF cycles, 3,421 were made in one clinic, while the remaining 258 were made in another. The distribution of infertility factors that led to treatment was male (29.8%), tubal (18.8%), endometriosis (7.1%), ovulatory (6.0%), diminished ovarian reserve (3.3%), male and female factors (19.1%), multiple female factors (5.5%), unexplained infertility (15.3%), and others (4.1%). Baseline characteristics and outcomes of the entire cohort, and separated by group are shown in table 1 .

**Table 1:** Baseline characteristics and outcomes of the entire cohort

	Freeze-all cycles (n=471)	Fresh cycles (n=3,208)	p value
Female age	34.37 (4.30)	35.40 (4.28)	<0.001
Diagnosis			<0.001
Unexplained	67 (14.2)	486 (15.1)	
Diminished ovarian reserve	30 (6.4)	95 (3.0)	
Chronic anovulation	37 (7.9)	185 (5.8)	
Endometriosis	37 (7.9)	224 (7.0)	
Tubal factor	64 (13.6)	626 (19.5)	
Male factor	119 (25.3)	976 (30.4)	
Multiple female	22 (4.7)	175 (5.4)	
Combined (female + male)	80 (17.0)	305 (9.5)	
Other	15 (3.2)	136 (4.2)	
Number of cleavage-stage embryos transferred	2.23 (0.60)	2.37 (0.73)	<0.001
Number of blastocysts transferred	1.54 (0.54)	1.89 (0.72)	<0.001
Clinical pregnancy rate	32.5 (152/467)	29.7 (952/3,208)	<0.001
Ongoing pregnancy rate	29.3 (137/467)	27.2 (872/3,208)	<0.001
Multiple pregnancy rate	25.7 (39/152)	27.4 (261/952)	0.723
Miscarriage rate	9.9 (15/152)	8.4 (80/952)	0.658
Implantation rate	22.8 (208/912)	17.4 (1,316/7,561)	0.024

For freeze-all cycles, the mean number of frozen embryos was 3.42 (SD, 3.46) for cleavage-stage embryos, and 1.65 (SD, 2.41) for blastocysts. The median interval between oocyte collection and thawing of embryos in the Freeze-all Group was 54 days, and the survival rate after warming was 97.14%. In four cases, no embryos survived after warming, resulting in 467 transfers actually performed in Freeze-all Group.

### Propensity score matching

**Figure 1:**
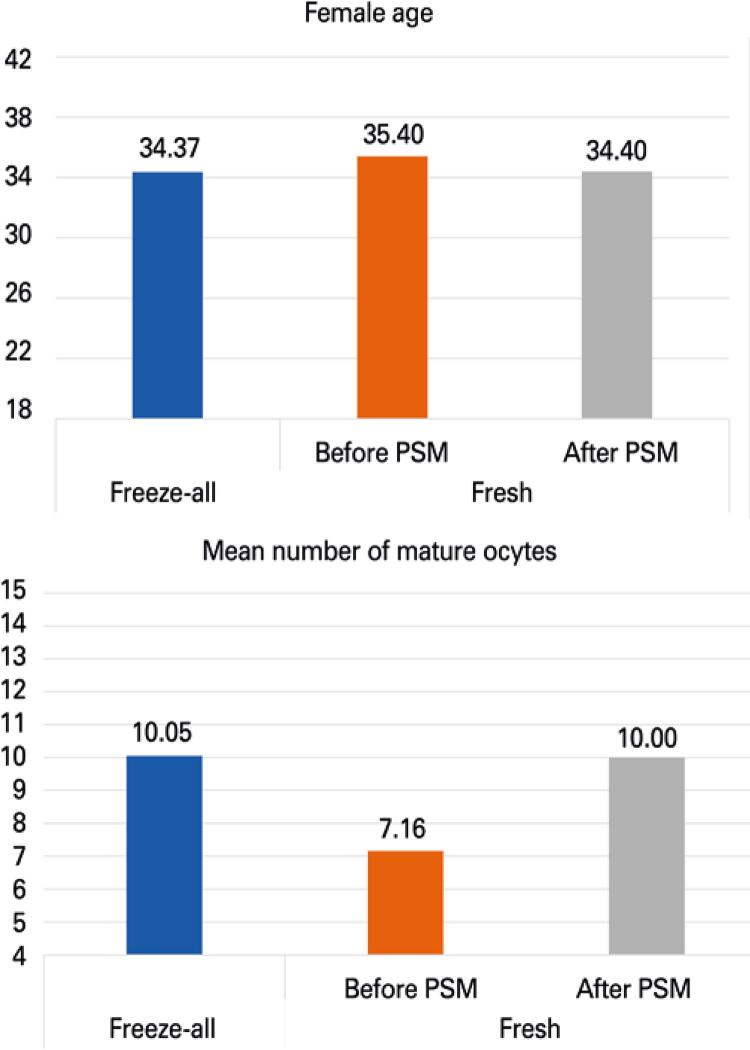
Bar graph of age and number of mature oocyte data for Freeze-all and Fresh Groups, before and after propensity score matching

Comparison of patient age and number of oocytes after propensity score matching is shown in figure 1 . This resulted in 467 and 934 matched cases in the freeze-all and Fresh Group, respectively (ratio of 1:2). Clinical outcomes after propensity score matching did not differ between the Fresh and Freeze-all Groups ( Table 2 ).

PSM: propensity score.

**Table 2:** Clinical outcomes after propensity score matching

	Freeze-all Group (n=467)	Fresh Group (n=934)	p value
Clinical pregnancy rate	32.5 (152/467)	32.3 (302/934)	0.936
Ongoing pregnancy rate	29.3 (137/467)	30.2 (254/934)	0.741
Multiple pregnancy rate	25.7 (39/152)	29.8 (90/302)	0.723
Miscarriage rate	9.9 (15/152)	6.6 (20/302)	0.224
Implantation rate	22.8 (208/912)	19.5 (413/2,120)	0.329

### Logistic regression analysis

Logistic regression analysis revealed the confounding variables with the greatest significance to be female age, number of mature oocytes collected, and quality of the embryo transferred. Patients aged 36 to 39 years and over 40 years were 20% (p=0.009) and 60% (p<0.001) less likely to have a clinical pregnancy, respectively. Regarding the number of mature oocytes, clinical pregnancy rate increased by 4% for each additional mature egg over four eggs (p=0.001). Patients that transferred good quality embryos were 86% more likely to have a clinical pregnancy (p<0.001).

**Figure 2:**
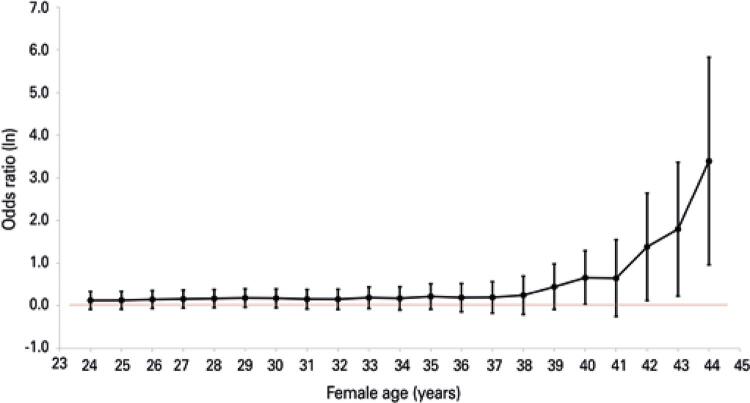
Clinical pregnancy rate comparing freeze-all and fresh transfer according to different female age cutoffs

In the multiple logistic regression model, two variables were associated with higher clinical pregnancy rate within the Freeze-all Group: age and stage at embryo transfer. When testing for many different age cutoffs ( Table 3 ), we found that for 623 women aged 40 years and older, the clinical pregnancy rate was significantly higher in the Freeze-all Group, as shown in figure 2 . Regarding the embryo stage at transfer, blastocyst transfer was associated with twice the chance of clinical pregnancy compared with cleavage embryos (OR=1.948, 95%CI: 1.219-3.114, p=0.005).

**Table 3:** Clinical pregnancy rate comparing freeze-all and fresh transfer, according to different female age cutoffs

Age cutoff (years)	Patients below the cutoff (n)	Patients above the cutoff (n)	Odds ratio (95%CI)
24	24	3,651	1.131 (0.917-1.3940)
25	38	3,637	1.135 (0.920-1.400)
26	58	3,617	1.155 (0.936-1.426)
27	93	3,582	1.169 (0.946-1.445)
28	143	3,532	1.182 (0.955-1.464)
29	242	3,433	1.199 (0.965-1.489)
30	357	3,318	1.189 (0.952-1.484)
31	493	3,182	1.165 (0.926-1.465)
32	685	2,990	1.162 (0.914-1.476)
33	927	2,748	1.206 (0.935-1.555)
34	1,210	2,465	1.188 (0.904-1.562)
35	1,528	2,147	1.241 (0.919-1.676)
36	1,879	1,796	1.209 (0.867-1.686)
37	2,175	1,500	1.215 (0.840-1.757)
38	2,479	1,196	1.278 (0.814-2.004)
39	2,789	886	1.561 (0.915-2.663)
40	3,052	623	1.931 (1.030-3.623)
41	3,280	395	1.911 (0.777-4.701)
42	3,454	221	3.980 (1.128-14.048)
43	3,562	113	6.019 (1.252-28.933)
44	3,622	53	29.764 (2.611-339.309)

Ln: napierian logarithm values not crossing the line are statistically significant (p<0.05).

## DISCUSSION

The present study analyzed clinical results of the freeze-all strategy for patients with normal response to ovarian stimulation in IVF cycles. In our cohort, the patients submitted to the freeze-all strategy were, on average, one year younger, and had three more eggs in comparison with women undergoing fresh transfer.

The 2015 report of Latin America Assisted Reproductive Techniques clearly states that female age is strongly associated with pregnancy rates in IVF treatments. For example, in women aged up to 30 years, the livebirth rate per transfer is 35%, while this rate decreases to less than 10% in women over 40 years.^( 15 )^ These results are in line with the findings of the present study.

Previous authors have shown that pregnancy rates following IVF increase progressively with the number of oocytes recovered. Sunkara et al., demonstrated for 35-year-old patients, the odds of livebirth are 22% when four oocytes are recovered, but 41% when 15 were recovered.^( 16 )^ Polyzos et al., reported that the chance of a livebirth with one cycle of transfer gradually increased for recovery of 4 to 7 oocytes, after which the rate plateaued until 20 oocytes.^( 17 )^ Patients with fresh transfers in our study were more commonly between 4 and 7 oocytes, and in Freeze-all Group, over 7 oocytes.

To minimize impact, we adjusted our data for age and number of oocytes using propensity score matching. This enabled partial correction of the selection bias, which was produced by the retrospective observational study design, by creating two comparable groups and simulating a randomization that is typical of prospective studies.^( 18 )^ Following this, statistical differences were not observed between the Freeze-all and Fresh Groups with regards to clinical and ongoing pregnancy rates. This finding agrees with two recent systematic reviews, which did not demonstrate the superiority of the freeze-all strategy for patients with normal response to ovarian stimulation.^( 12 , 13 )^

Our analysis of the stage at which embryos were transferred indicated a clinically relevant and statistically significant higher pregnancy rate, when thawed blastocysts were transferred in freeze-all cycles. Previous studies evolving high responder patients indicated extended culture and blastocyst freezing were conducive to improved results with the freeze-all strategy, mainly after the widespread utilization of vitrification technique.^( 19 - 23 )^

Regarding the woman’s age, we found a statistically significant higher clinical pregnancy rate in the Freeze-all Group for patients 40 years of age and older, even when adjusted for number of oocytes and stage of embryos transferred. Although our study does not allow us to know the causes that led to this finding, we think that one possible explanation would be inability of the embryos from older patients – in theory with lower implantation potential – to supplant lower endometrial receptivity of the fresh cycle.^( 24 )^ Another possibility is the existence of hidden confounding variables not evaluated in this study. For example, a selection bias that may have led to the freeze-all strategy for patients with a premature progesterone rise, more common in older patients during controlled ovarian stimulation.^( 25 , 26 )^ Previous studies have demonstrated the correlation between elevated serum progesterone levels on the day of ovulation trigger, and lower pregnancy rates in fresh cycles.^( 27 , 28 )^

Regardless of the cause, we considered that our cohort of normo-responder women aged 40 years and over is large and, to the best of our knowledge, this is the first study showing an association between freeze-all strategy and higher clinical pregnancy rate for this group of patients. To further elucidate it, the next step should be to perform a prospective randomized controlled trial involving patients aged 40 years and over, with normal response to ovarian stimulation.

This study had some other limitations which should be acknowledged. First, just like every retrospective study, it suffered from selection bias; patients in the Freeze-all Group were younger and had more oocytes than patients in the fresh-embryo transfer group. It was partially corrected using propensity score matching, as stated above. Second, we were unable to obtain data regarding the reasons for choosing the freeze-all strategy ( *e.g.* , a progesterone elevation at trigger day, a high risk of ovarian hyperstimulation syndrome). In addition, we did not have detailed data about the endometrial preparation protocols used, or the reason for choosing different ovulation triggers during ovarian stimulation. However, we think that these weaknesses can be mitigated, as reported in recent Cochrane systematic reviews, which have studied these variables and demonstrated no difference in clinical pregnancy rates in thawed embryo transfer cycles.^( 29 , 30 )^

## CONCLUSION

In conclusion, our study showed the freeze-all policy results in similar pregnancy rates as fresh transfer for patients with normal response to ovarian stimulation, after adjustment for age and the number of oocytes retrieved. For patients aged 40 years and older, however, the freeze-all strategy was associated with higher clinical pregnancy rates. This deserves further investigation in future research.
