# Supplementary material for: Freeze-all policy for in vitro fertilization in women with normal response to ovarian stimulation
Source: Einstein (Sao Paulo). 2021 Sep 30;19:eAO6290. doi: 10.31744/einstein_journal/2021AO6290 (PMC8483637; doi:10.31744/einstein_journal/2021AO6290)
Supplement: Translation

# Translation: Congelamento de todos os embriões em ciclos de fertilização *in vitro* em mulheres com resposta normal à estimulação ovariana

**Authors:** Oscar Barbosa Duarte-Filho, Sérgio Podgaec

**Affiliations:** 1 Brasil Hospital Israelita Albert Einstein, São Paulo, SP, Brasil

**Keywords:** Fertilização *in vitro*, Indução da ovulação, Transferência embrionária, Criopreservação, Vitrificação

## Abstract

**Objetivo:**

Responder à pergunta se a estratégia *freeze-all* (congelamento de todos os embriões) e subsequente transferência de embriões congelados é preferível à transferência de embriões a fresco em pacientes com resposta normal à estimulação ovariana (4 a 15 ovócitos coletados) durante tratamentos de fertilização *in vitro* .

**Métodos:**

Coorte retrospectiva de dois centros de reprodução humana entre 2013 e 2017. No total, foram incluídas 471 transferências de ciclos com congelamento de todos os embriões, e 3.208 transferências a fresco.

**Resultados:**

Após o ajuste do escore de propensão para idade e número de óvulos, foram analisados 467 ciclos com congelamento de todos os embriões e 934 ciclos a fresco, não havendo diferença estatisticamente significativa entre os grupos em relação à taxa de gravidez clínica (32,5% no Grupo *Freeze-all* e 32,3% no Grupo a Fresco, p=0,936). Para mulheres com 40 anos ou mais, observamos uma taxa de gravidez clínica estatisticamente maior quando foi utilizada a estratégia *freeze-all* (29,3% no Grupo *Freeze-all* e 19,8% no Grupo a Fresco, p=0,04).

**Conclusão:**

A estratégia *freeze-all* não foi superior à transferência a fresco para todas as pacientes com resposta normal à estimulação ovariana. No entanto, mulheres com 40 anos ou mais podem ter algum benefício com essa estratégia. Isso justifica uma investigação mais aprofundada em pesquisas futuras e, de preferência, em estudos prospectivos randomizados.

## INTRODUÇÃO

O primeiro nascimento humano produzido por fertilização *in vitro* (FIV) ocorreu a partir de uma transferência de embrião a fresco, na Inglaterra, em 1978.^( 1 )^Cinco anos depois, um grupo australiano registrou o primeiro nascimento após transferência de embrião congelado.^( 2 )^Desde então, mais de sete milhões de bebês já nasceram por meio de FIV, com transferência de embriões a fresco ou congelados.^( 3 )^

No princípio da era da FIV, a transferência de embriões a fresco era o padrão, porque os resultados após congelamento dos embriões usando a técnica de congelamento lento eram insatisfatórios.^( 4 )^A transferência de embriões congelados ficava restrita aos embriões excedentes, ou caso a transferência a fresco não fosse possível, o que ocorria principalmente em pacientes com alta resposta à estimulação ovariana controlada, e com alto risco de síndrome da hiperestimulação.^( 5 )^

Na última década, o uso de embriões descongelados aumentou significativamente. Um estudo online recente, envolvendo especialistas de vários países, revelou que quase 85% das clínicas incluídas no estudo oferecem rotineiramente transferência de embriões congelados para as pacientes.^( 6 )^

Dois fatos mudaram a prática de transferências a fresco: a demonstração de que a colocação de embriões a fresco no endométrio, sob efeito dos medicamentos usados na estimulação ovariana pode alterar a receptividade do endométrio,^( 7 )^e o desenvolvimento de métodos de vitrificação de embriões humanos - congelamento ultra-rápido, mais simples, com melhor sobrevida, e maiores taxas de gravidez que a técnica de congelamento lento.^( 8 )^Esses fatores levaram a um debate crescente sobre se o padrão de tratamento deve mudar da atual estratégia de “ *freeze-all* para pacientes selecionadas” para uma estratégia de “ *freeze-all* para todas as pacientes”.^( 9 - 11 )^

Duas metanálises recentes encontraram taxas de gravidez semelhantes entre as duas estratégias para pacientes com resposta normal à estimulação ovariana, mas há uma grande heterogeneidade entre os estudos, e as evidências que corroboram a estratégia estudada ainda são consideradas de baixa qualidade.^( 12 , 13 )^

## OBJETIVO

Comparar as taxas de gravidez clínica da transferência de embriões a fresco e transferência de embriões congelados em ciclos *freeze-all* de pacientes com resposta normal à estimulação ovariana, e identificar nesse grupo de pacientes as variáveis clínicas associadas a maiores taxas de gravidez dentro de cada estratégia.

## MÉTODOS

### Pacientes e ciclos

Trata-se de um estudo retrospectivo, incluindo dados extraídos de prontuários eletrônicos de todos os ciclos autólogos de FIV, realizados nas clínicas Projeto ALFA e Vidabemvinda, São Paulo, Brasil, entre janeiro de 2013 e dezembro de 2017, nos quais foram coletados 4 a 15 ovócitos maduros (o que foi considerado resposta normal). Apenas a primeira transferência do Grupo a Fresco e o primeiro descongelamento do Grupo *Freeze-all* foram considerados para análise. Os critérios de exclusão foram ciclos de punção de óvulos sem descongelamento de embriões para transferência dentro do período do estudo; pacientes envolvidas no programa de doação de ovócitos, transferências de embriões acumulados de outras punções de óvulos; ou ciclos submetidos a testes genéticos pré-implantação (congelamento de todos os embriões obrigatório em ambas as clínicas). O estudo e todos os seus protocolos foram aprovados pelo Comitê de Ética do Hospital Israelita Albert Einstein (HIAE), em São Paulo (Brasil), no momento da coleta de dados (número de aprovação: 2.506.239, CAAE: 79213617.1.3001.5670) e do Hospital Santa Paula (número de aprovação: 2.506.239, CAAE: 79213617.1.3001.5670), e isentos da obrigação de coletar consentimento livre e esclarecido. A confidencialidade e integridade dos dados foram mantidas e a identidade dos pacientes foi preservada.

### Protocolo de estimulação e preparação do endométrio

A estimulação ovariana controlada foi realizada com hormônio folículo-estimulante recombinante, menotropina, ou uma combinação de ambos. Um antagonista do hormônio liberador de gonadotrofina (GnRH) foi usado para supressão da hipófise em mais de 90% dos ciclos. Em todos os casos com transferência a fresco, o gatilho incluiu o uso de gonadotrofina coriônica humana (hCG) recombinante ou urinária, isoladamente ou em combinação com agonistas do GnRH, a critério do médico. A preparação do endométrio para transferência de embriões congelados foi feita com estradiol e progesterona ou com ciclo natural, a critério do médico e da paciente.

### Cultura e criopreservação dos embriões

Todos os casos fizeram injeção intracitoplasmática de espermaotzoides ou FIV convencional. Os embriões permaneceram em cultura até a transferência na fase de clivagem (segundo ou terceiro dia) ou blastocisto (quinto ou sexto dia), e foram classificados de acordo com a descrição da *Society for Assisted Reproductive Technology* (SART).^( 14 )^No Grupo *Freeze-all* , todos os embriões foram congelados na fase de clivagem ou blastocisto, usando-se o método de vitrificação com dois kits comerciais de vitrificação (Cryotop^®^Vitrification, Kitazato, Japão; Vitringa^®^, Ingamed, Brasil).

### Variáveis e desfechos

As variáveis independentes analisadas foram idade da mulher, número de ovócitos e número de ovócitos maduros, taxa de fertilização, número de zigotos, número e qualidade dos embriões em clivagem e blastocistos, e número e estágio dos embriões transferidos.

O desfecho primário foi a gravidez clínica, definida como visualização do saco gestacional no útero à ultrassonografia. Quando mais de um saco gestacional foi visto, a gravidez foi considerada múltipla. A taxa de implantação foi calculada dividindo-se o número de sacos gestacionais pelo número de embriões transferidos. Aborto foi definido como interrupção da gravidez após a visualização do saco gestacional no útero.

### Análise estatística

Verificou-se a normalidade das variáveis quantitativas por meio de histogramas, diagramas de caixa e gráficos de quantis. Para a variável quantitativa normal, avaliamos as médias e desvios padrão (DP) e utilizamos o teste *t* de Student para comparar as médias. Para dados não distribuídos normalmente, usamos a mediana e a faixa interquartil, e empregamos o teste de Mann Whitney para comparações. Para as variáveis qualitativas, são apresentados o número e a proporção de observações em cada categoria, e os dados foram comparados pelo teste χ^2^ou teste exato de Fisher. Para avaliar o efeito das estratégias a fresco e *freeze-all* sobre os resultados de interesse, controlando para as demais variáveis, usamos modelos lineares generalizados da família binomial com regressão logística (modelos de regressão logística).

Avaliamos o balanço de amostras do banco de dados completo, calculando diferenças padronizadas e utilizando o teste χ^2^Omnibus. Com base nos resultados da regressão logística simples, pareamos os grupos utilizando o método do escore de propensão, considerando duas covariáveis: número de óvulos maduros e idade da mulher. Assim, pareamos os casos na proporção de 1:2 dos ciclos nos Grupos *Freeze-all* e a Fresco. Essa seleção de casos foi realizada pelo método do vizinho mais próximo ( *nearest-neighbor* ); para cada transferência no Grupo *Freeze-all* , selecionamos duas transferências no Grupo a Fresco, com o escore de propensão mais próximo. Os resultados das transferências em fase de clivagem e blastocisto foram analisados separadamente.

## RESULTADOS

### Características da coorte

Este estudo incluiu 3.679 ciclos de FIV: 471 que usaram a estratégia *freeze-all* e 3.208 transferências a fresco. Desses ciclos de FIV, 3.421 foram realizados em uma clínica, e os restantes 258 foram realizados em outra. A distribuição dos fatores de infertilidade que levaram ao tratamento foi masculinos (29,8%), tubários (18,8%), endometriose (7,1%), ovulatórios (6,0%), baixa reserva ovariana (3,3%), fatores masculinos e femininos (19,1%), múltiplos fatores femininos (5,5%), infertilidade inexplicada (15,3%), e outros (4,1%). As características iniciais, os desfechos de toda a coorte, e os resultados separados por grupos são mostrados na tabela 1 .

**Tabela 1:** Características iniciais e desfechos de toda a coorte

	Com congelamento de todos os embriões (n=471)	Ciclos a fresco (n=3.208)	Valor de p
Idade da mulher	34,37 (4,30)	35,40 (4,28)	<0,001
Diagnóstico			<0,001
Não explicado	67 (14,2)	486 (15,1)	
Baixa reserva ovariana	30 (6,4)	95 (3,0)	
Anovulação crônica	37 (7,9)	185 (5,8)	
Endometriose	37 (7,9)	224 (7,0)	
Fator tubário	64 (13,6)	626 (19,5)	
Fator masculino	119 (25,3)	976 (30,4)	
Múltiplos fatores femininos	22 (4,7)	175 (5,4)	
Combinação (mulher + homem)	80 (17,0)	305 (9,5)	
Outros	15 (3,2)	136 (4,2)	
Número de embriões em estágio de clivagem transferidos	2,23 (0,60)	2,37 (0,73)	<0,001
Número de blastocistos transferidos	1,54 (0,54)	1,89 (0,72)	<0,001
Taxa de gravidez clínica	32,5 (152/467)	29,7 (952/3.208)	<0,001
Taxa de gravidez em curso	29,3 (137/467)	27,2 (872/3.208)	<0,001
Taxa de gravidez múltipla	25,7 (39/152)	27,4 (261/952)	0,723
Taxa de aborto espontâneo	9,9 (15/152)	8,4 (80/952)	0,658
Taxa de implantação	22,8 (208/912)	17,4 (1.316/7.561)	0,024

Para os ciclos *freeze-all* , o número médio de embriões congelados foi 3,42 (DP 3,46) para fase de clivagem, e 1,65 (DP 2,41) para blastocistos. O intervalo mediano entre a punção dos ovócitos e o descongelamento dos embriões no Grupo *Freeze-all* foi de 54 dias, e a taxa de sobrevida após o descongelamento foi de 97,14%. Em quatro casos, nenhum embrião sobreviveu após o descongelamento, portanto, foram efetivamente realizadas 467 transferências no Grupo *Freeze-all* .

### Pareamento por escore de propensão

A comparação entre a idade da paciente e o número de ovócitos após o pareamento por escore de propensão é apresentada na figura 1 . O resultado foi 467 e 934 casos pareados nos Grupos *Freeze-all* e a Fresco, respectivamente (razão de 1:2). Os resultados clínicos após o pareamento por escore de propensão não diferiram entre os Grupos a Fresco e *Freeze-all* ( Tabela 2 ).

**Figura 1:** Gráfico de barras da idade e número de ovócitos maduros para os Grupos *Freeze-all* e Fresco, antes e depois do pareamento por escore de propensão

PSM: pareamento por escore de propensão.

**Tabela 2:** Resultados clínicos após o pareamento por escore de propensão

	Grupo *Freeze-all* (n=467)	Grupo a Fresco (n=934)	Valor de p
Taxa de gravidez clínica	32,5 (152/467)	32,3 (302/934)	0,936
Taxa de gravidez em curso	29,3 (137/467)	30,2 (254/934)	0,741
Taxa de gravidez múltipla	25,7 (39/152)	29,8 (90/302)	0,723
Taxa de aborto espontâneo	9,9 (15/152)	6,6 (20/302)	0,224
Taxa de implantação	22,8 (208/912)	19,5 (413/2.120)	0,329

### Análise de regressão logística

A análise de regressão logística revelou as variáveis confundidoras de maior significância como sendo a idade da mulher, o número de ovócitos maduros puncionados, e a qualidade do embrião transferido. Pacientes com idade entre 36 e 39 anos e acima de 40 anos tiveram 20% (p=0,009) e 60% (p<0,001) menos probabilidade de apresentar gravidez clínica, respectivamente. Em relação ao número de ovócitos maduros, a taxa de gravidez clínica aumentou 4% para cada óvulo maduro adicional acima de quatro óvulos (p=0,001). Pacientes que transferiram embriões de boa qualidade tiveram probabilidade 86% maior de gravidez clínica (p<0,001).

No modelo de regressão logística múltipla, duas variáveis foram associadas à maior taxa de gravidez clínica no Grupo *Freeze-all* : idade e estágio embrionário no momento da transferência. Ao testar diversos pontos de corte para idade ( Tabela 3 ), verificamos que, para 623 mulheres com idade igual ou superior a 40 anos, a taxa de gravidez clínica foi significativamente maior no Grupo *Freeze-all* , como mostra a figura 2 . Em relação ao estágio embrionário no momento da transferência, a transferência de blastocisto foi associada ao dobro da probabilidade de gravidez clínica em comparação aos embriões em fase de clivagem (OR=1,948; IC95%: 1,219-3,114; p=0,005).

**Tabela 3:** Taxa de gravidez clínica comparando a transferência de embriões congelados ( *freeze-all* ) e a fresco, de acordo com diferentes pontos de corte para idade da mulher

Limite de idade (anos)	Pacientes abaixo do limite (n)	Pacientes acima do limite (n)	*Odds ratio* (IC95%)
24	24	3.651	1,131 (0,917-1,3940)
25	38	3.637	1,135 (0,920-1,400)
26	58	3.617	1,155 (0,936-1,426)
27	93	3.582	1,169 (0,946-1,445)
28	143	3.532	1,182 (0,955-1,464)
29	242	3.433	1,199 (0,965-1,489)
30	357	3.318	1,189 (0,952-1,484)
31	493	3.182	1,165 (0,926-1,465)
32	685	2.990	1,162 (0,914-1,476)
33	927	2.748	1,206 (0,935-1,555)
34	1.210	2.465	1,188 (0,904-1,562)
35	1.528	2.147	1,241 (0,919-1,676)
36	1.879	1.796	1,209 (0,867-1,686)
37	2.175	1.500	1,215 (0,840-1,757)
38	2.479	1.196	1,278 (0,814-2,004)
39	2.789	886	1,561 (0,915-2,663)
40	3.052	623	1,931 (1,030-3,623)
41	3.280	395	1,911 (0,777-4,701)
42	3.454	221	3,980 (1,128-14,048)
43	3.562	113	6,019 (1,252-28,933)
44	3.622	53	29,764 (2,611-339,309)

**Figura 2:** Taxa de gravidez clínica comparando a transferência de embriões congelados ( *freeze-all* ) e a fresco, de acordo com diferentes pontos de corte para idade da mulher

Ln: os valores do logaritmo napieriano que não cruzam a linha são estatisticamente significativos (p<0,05).

## DISCUSSÃO

Este estudo analisou os resultados clínicos da estratégia *freeze-all* para pacientes com resposta normal à estimulação ovariana em ciclos de FIV. Em nossa coorte, as pacientes submetidas à estratégia *freeze-all* eram, em média, um ano mais jovens, e tiveram três óvulos a mais comparadas às pacientes submetidas a transferência a fresco.

O relatório *Latin America Assisted Reproductive Techniques* de 2015 afirma claramente que a idade da mulher tem forte associação às taxas de gravidez nos casos de FIV. Por exemplo, em mulheres com até 30 anos, a taxa de nascidos vivos por transferência é de 35%, mas essa taxa diminui para menos de 10% em mulheres com mais de 40 anos.^( 15 )^Esses resultados estão de acordo com os achados deste estudo.

Autores anteriores demonstraram que as taxas de gravidez após FIV aumentam progressivamente com o número de ovócitos puncionados. Sunkara et al. demonstraram que, para pacientes de 35 anos, a probabilidade de nascidos vivos é de 22% quando são puncionados 4 ovócitos, e 41% quando são puncionados 15 ovócitos.^( 16 )^Polyzos et al. relataram que a probabilidade de nascidos vivos com um ciclo de transferência aumentou gradualmente quando foram puncionados 4 a 7 ovócitos e, a partir deste ponto, ocorreu um platô até 20 ovócitos.^( 17 )^Pacientes com transferências a fresco em nosso estudo ficaram mais frequentemente entre 4 e 7 ovócitos, e no Grupo *Freeze-all* , ficaram acima de 7 ovócitos.

Para minimizar o impacto, ajustamos nossos dados para idade e número de ovócitos, usando o pareamento por escore de propensão. Isso permitiu a correção parcial do viés de seleção, produzido pelo desenho observacional retrospectivo, criando dois grupos comparáveis e simulando uma randomização típica de estudos prospectivos.^( 18 )^Depois disso, não foram observadas diferenças estatísticas entre os Grupos *Freeze-all* e Fresco em relação às taxas de gravidez clínica e em curso. Este achado está de acordo com duas revisões sistemáticas recentes, que não demonstraram superioridade da estratégia *freeze-all* para pacientes com resposta normal à estimulação ovariana.^( 12 , 13 )^

Nossa análise do estágio em que os embriões foram transferidos indicou aumento clinicamente relevante e estatisticamente significativo da taxa de gravidez, quando se transferiram blastocistos descongelados em ciclos *freeze-all* . Estudos prévios em pacientes com alta resposta indicaram que a cultura estendida e o congelamento de blastocistos levaram a melhores resultados com a estratégia *freeze-all* , principalmente após o uso generalizado da técnica de vitrificação.^( 19 - 23 )^

Quanto à idade da mulher, encontramos um aumento estatisticamente significativo na taxa de gravidez clínica do Grupo *Freeze-all* , em pacientes de 40 anos ou mais, mesmo quando ajustada para o número de ovócitos e o estágio dos embriões transferidos. Embora nosso estudo não nos permita saber as causas que levaram a esse achado, acreditamos que uma possível explicação seria a incapacidade dos embriões de pacientes mais velhas – teoricamente, com menor potencial de implantação – de superar a receptividade mais baixa do endométrio em um ciclo a fresco.^( 24 )^Outra possibilidade é a existência de variáveis confundidoras ocultas, não avaliadas neste estudo. Por exemplo, um viés de seleção, que pode ter levado à escolha da estratégia *freeze-all* para pacientes com aumento prematuro da progesterona, mais comum entre pacientes mais velhas durante a estimulação ovariana controlada.^( 25 , 26 )^Estudos anteriores demonstraram correlação entre níveis séricos elevados de progesterona no dia do gatilho da ovulação, e menores taxas de gravidez em ciclos a fresco.^( 27 , 28 )^

Independentemente da causa, consideramos que nossa coorte de mulheres com resposta normal, de 40 anos ou mais, é grande e, até onde sabemos, este é o primeiro estudo demonstrando associação entre a estratégia *freeze-all* e uma maior taxa de gravidez clínica nesse grupo de pacientes. Para entender isso melhor, o próximo passo deve ser a realização de um estudo prospectivo randomizado e controlado, envolvendo pacientes de 40 anos ou mais, com resposta normal à estimulação ovariana.

Este estudo teve algumas outras limitações que devem ser reconhecidas. Em primeiro lugar, assim como todos os estudos retrospectivos, houve viés de seleção; pacientes do Grupo *Freeze-all* eram mais jovens e tinham mais ovócitos que as pacientes do grupo de transferência de embriões a fresco. Isso foi parcialmente corrigido usando-se o pareamento por escore de propensão, como indicado acima. Em segundo lugar, não foi possível obter dados sobre as razões para a escolha da estratégia *freeze-all* (por exemplo, progesterona elevada no dia do gatilho, alto risco de síndrome de hiperstimulação ovariana). Além disso, não tínhamos dados detalhados sobre os protocolos de preparação endometrial usados, ou a razão para a escolha dos diferentes gatilhos de ovulação durante a estimulação ovariana. No entanto, acreditamos que essas deficiências podem ser atenuadas, conforme mostrado em revisões sistemáticas recentes da Cochrane, que investigaram essas variáveis e não mostraram diferença nas taxas de gravidez clínica em ciclos de transferência de embriões descongelados.^( 29 , 30 )^

## CONCLUSÃO

Em conclusão, nosso estudo mostrou que a política de congelamento de todos os embriões ( *freeze-all* ) resultou em taxas de gravidez semelhantes às da transferência a fresco em pacientes com resposta normal à estimulação ovariana, após ajuste para idade e número de ovócitos puncionados. Para as pacientes com 40 anos ou mais, no entanto, a estratégia *freeze-all* foi associada a maiores taxas de gravidez clínica. Isso justifica uma investigação mais aprofundada no em pesquisas futuras.
